# OP07 Shared Learning: An Alternative Model For Patient Capacity Development To Participate In Health Technology Assessment

**DOI:** 10.1017/S0266462325100664

**Published:** 2025-12-29

**Authors:** Kris Pierce, Ann Single, Melanie Funk, Karen Van Gorp

## Abstract

**Introduction:**

The Shared Learning model redefines patient engagement in health technology assessment (HTA) by combining immersion, knowledge sharing, and network expansion. Through flexible programs like Patient Voice Initiative (PVI) Patient Partners, it empowers advocates to deepen their understanding of HTA processes and disseminate insights broadly. This innovative, scalable approach ensures patient voices are central to decision-making, fostering informed contributions and addressing the challenges of rapidly evolving health technologies.

**Methods:**

The Shared Learning model piloted by the PVI supported three patient advocates through a structured immersion in HTA processes. Advocates engaged directly in HTA discussions, meetings, and consultations, attending the HTAi Annual Meeting 2024 for deeper insights and networking. The model emphasized peer knowledge dissemination through webinars, workshops, and community forums, creating a ripple effect of shared learning. Advocates reflected on their experiences, identifying insights to benefit broader patient networks. This approach combined immersive learning, knowledge-building, and capacity expansion, fostering both vertical engagement with HTA stakeholders and horizontal knowledge-sharing within patient communities.

**Results:**

The Shared Learning pilot demonstrated success in enhancing patient engagement within HTA. Advocates deepened their understanding of HTA processes through direct participation and shared insights with wider patient networks, fostering a multiplier effect. Peer-to-peer knowledge dissemination expanded patient capacity, strengthening advocacy communities. Advocates utilized their experiences to host webinars, workshops, and community forums, broadening the reach of HTA knowledge. The initiative also improved the ability of patient networks to contribute meaningfully to decision-making processes. These outcomes highlight the model’s potential for creating sustainable, informed, and resilient advocacy communities equipped to address the challenges of rapidly evolving health technologies.

**Conclusions:**

The Shared Learning model revolutionized patient engagement in HTA by emphasizing immersion, knowledge sharing, and network expansion. Through flexible programs like PVI Patient Partners, the model deepened engagement while enabling broader dissemination of insights. As health technologies rapidly evolve, this scalable approach ensures patient voices remain central to HTA decision-making, fostering informed, sustainable contributions within patient communities.

